# 3D-Assisted Quantitative Assessment of Orbital Volume Using an Open-Source Software Platform in a Taiwanese Population

**DOI:** 10.1371/journal.pone.0119589

**Published:** 2015-03-16

**Authors:** Victor Bong-Hang Shyu, Chung-En Hsu, Chih-hao Chen, Chien-Tzung Chen

**Affiliations:** 1 Craniofacial Research Center, Department of Plastic and Reconstructive Surgery, Chang Gung Memorial Hospital at Linkou, Chang Gung University, College of Medicine, Taoyuan, Taiwan; 2 Department of Medicine, College of Medicine, Chang Gung University, Taoyuan, Taiwan; 3 Department of Plastic and Reconstructive Surgery, Chang Gung Memorial Hospital at Keelung, Keelung, Taiwan; Sun Yat-sen University, CHINA

## Abstract

Orbital volume evaluation is an important part of pre-operative assessments in orbital trauma and congenital deformity patients. The availability of the affordable, open-source software, OsiriX, as a tool for preoperative planning increased the popularity of radiological assessments by the surgeon. A volume calculation method based on 3D volume rendering-assisted region-of-interest computation was used to determine the normal orbital volume in Taiwanese patients after reorientation to the Frankfurt plane. Method one utilized 3D points for intuitive orbital rim outlining. The mean normal orbital volume for left and right orbits was 24.3±1.51 ml and 24.7±1.17 ml in male and 21.0±1.21 ml and 21.1±1.30 ml in female subjects. Another method (method two) based on the bilateral orbital lateral rim was also used to calculate orbital volume and compared with method one. The mean normal orbital volume for left and right orbits was 19.0±1.68 ml and 19.1±1.45 ml in male and 16.0±1.01 ml and 16.1±0.92 ml in female subjects. The inter-rater reliability and intra-rater measurement accuracy between users for both methods was found to be acceptable for orbital volume calculations. 3D-assisted quantification of orbital volume is a feasible technique for orbital volume assessment. The normal orbital volume can be used as controls in cases of unilateral orbital reconstruction with a mean size discrepancy of less than 3.1±2.03% in females and 2.7±1.32% in males. The OsiriX software can be used reliably by the individual surgeon as a comprehensive preoperative planning and imaging tool for orbital volume measurement and computed tomography reorientation.

## Introduction

Quantitative determination of orbital volume is valuable to the evaluation and management of many conditions affecting the orbit. The structure of the orbit can be influenced by various diseases, such as intraorbital tumors (e.g. adenoid cystic carcinoma, retinoblastoma), inflammatory etiologies (e.g. sarcoidosis, Grave’s disease), congenital diseases (e.g. congenital orbital dysplasia, Pfeiffer syndrome), and traumatic orbital fractures. Noticeable manifestations of alterations in the orbital structure include physical signs and symptoms such as exophthalmos, enophthalmos, hypophthalmus, and diplopia.

For reconstructive plastic surgeons, the primary concern lies with the bony structure of the orbit. The principal goal of surgical intervention in trauma or congenital deformity is restoration of the bony anatomy of the orbital cavity. This should result in correction of globe position and assist in correcting visual manifestations such as diplopia [1]. In the past, empirical assessments formed the basis of decision-making within the operating room. However, this often results in over or under-correction, and clinical experience demonstrates that globe position post-operatively is highly unpredictable [2].

Computed tomography (CT) based methodologies have been used since the 1980s to assist in formulating surgical plans with higher levels of accuracy [3]. Recently, advancements in CT have led to images with higher resolution and less noise. 3D technologies such as volume/surface rendering and region-of-interest (ROI) based volume computations provide additional information to traditional 2D CT images. For example, mastication muscle volume was evaluated by Analyze software to determine the influence of osseus mandible versus muscle in patients with square-face [4]. CT volumetry was also assessed as a pre-operative planning tool to evaluate residual liver volume in hepatic carcinoma surgery [5]. Apparently, the ability to accurately determine orbital volume could provide useful information in orbital reconstruction. However, the information obtained from normal population needs to be completed before executing volumetric assessments in diseased orbits.

Apparently, differences exist in orbital volume among ethnic groups [6–9], with there being insufficient information describing the normal orbital volume in Taiwanese adults. One purpose of this study was to perform a leading volumetric analysis of normal orbits in Taiwanese adult patients using 3D imaging software based on CT data. An open-source platform for personal computers, OsiriX, was used, with the second aim of establishing a convenient pre-operative planning process for individual surgeons. A novel 3D-assisted methodology was used to perform calculations on orbital volume that provided a more intuitive method of evaluation for the non-radiologist. In order to clarify the difference between two anterior limit definitions of orbital volume that exists in the literature, two different methods were compared using OsiriX software [7, 10, 11]. Finally, the inter-rater and intra-rater variability was compared to validate the accuracy of this tool through statistical analysis.

## Subjects and Methods

### Subjects

Twenty Taiwanese adults (10 male and 10 female) were randomly selected from the patients examined at Chang Gung Memorial Hospital, Linkou Branch between January and December, 2011. The mean chronological age was 30±12 years old (range 16 to 57). Only cases with bilateral normal orbits were included. All patients had received facial bone CT scans for evaluating craniofacial deformities other than conditions affecting the orbit, or trauma surveys with negative results. Exclusion criteria included underlying conditions such as congenital craniofacial malformations, cleft palate, thyroid diseases, previous orbital or eye surgery, and history of orthodontic or orthognathic surgery. Only patients older than 16 years old were included as this is the reported age for cessation of orbital volume increase [6, 8]. The study was approved by the Institutional Review Board of Chang Gung Memorial Hospital, Linkou Branch and adhered to ethical guidelines. Due to the retrospective nature of the study, patient consent was not required after ethical committee approval of the study for both minors and adults. Written consent was not obtained from participants for records usage, and clinical records were anonymized and de-identified prior to analysis.

### CT data acquisition

Computed tomography data was acquired on a 16-row multi-slice CT (Siemens Sensation 16, Siemens, Forchheim, Germany) using a high-resolution facial bone protocol for adult patients. Protocol details are Tube voltage, 120 kvp; Tube current, 67 mA; Slice thickness, 1mm; Slice distance, 0.8 mm; Gantry tilt: 0°

Position, Head-first Supine; Matrix, 512 x 512. For image analysis, files were exported in DICOM format via compact disc to an offline Macbook Pro workstation (Apple, Cupertino, CA, USA) with specifications: CPU, 2.4Ghz Intel Core i7; Memory, 8GB DDR3; GPU, 1024 MB AMD Radeon HD 6770M; Operating System, OS X 10.8.4.

### Orbital volume determination

OsiriX MD (FDA cleared, Pixmeo) was used for image processing and analysis. Two methods that differed in the anterior limit determination were applied for evaluation of orbital volume under bone window. A reconstructed Frankfurt plane was used for manual ROI segmentation. Frankfurt plane is also known as auriculo-orbital plane, and is defined as the plane passing through the upper margin of the external auditory meatus and the inferior margin of the orbit. Two methods reported in the literature were utilized to calculate the orbital volume. Method one utilizes a 3D-assisted methodology and corresponds anatomically to the gold standard method of fluid displacement volume measurement, while method two is a variation reported in the literature to serve as a comparison. The methods are described in the Results section.

### Data analysis

The left and right orbital volumes were calculated individually using both method one (n = 20) and method two (n = 20) by a surgeon familiar with the OsiriX platform (Rater 1). To evaluate if the methodologies and software could produce reproducible results between users, the left orbit was re-evaluated by another surgeon new to the software (Rater 2) but familiar with orbital anatomy (n = 10). The rules for orbital volume calculation and anatomical landmarks were provided, and the results were compared. To assess the reproducibility by the same user, the left orbit was re-evaluated 2 weeks after initial assessment by Rater 1 (n = 10).

### Statistical methods

Statistical data is reported as mean ± standard deviation. Pearson’s correlation coefficient was used to evaluate the correlation and relationship between left and right orbital volume. The mean difference between left and right orbits for patients was reported to further illustrate the relationship. Unpaired *t*-test was used to compare the orbital volume results between genders. Unpaired t-test and mean difference was used to compare the difference between method one and two. Bland-Altman plots were also used to describe the reliability between raters using method one and method two, and the relationship between method one and two [12]. Sigmaplot 12.5 (Systat Software, U.K.) was used for graphing and statistical analysis.

## Results

**Fig 1:**
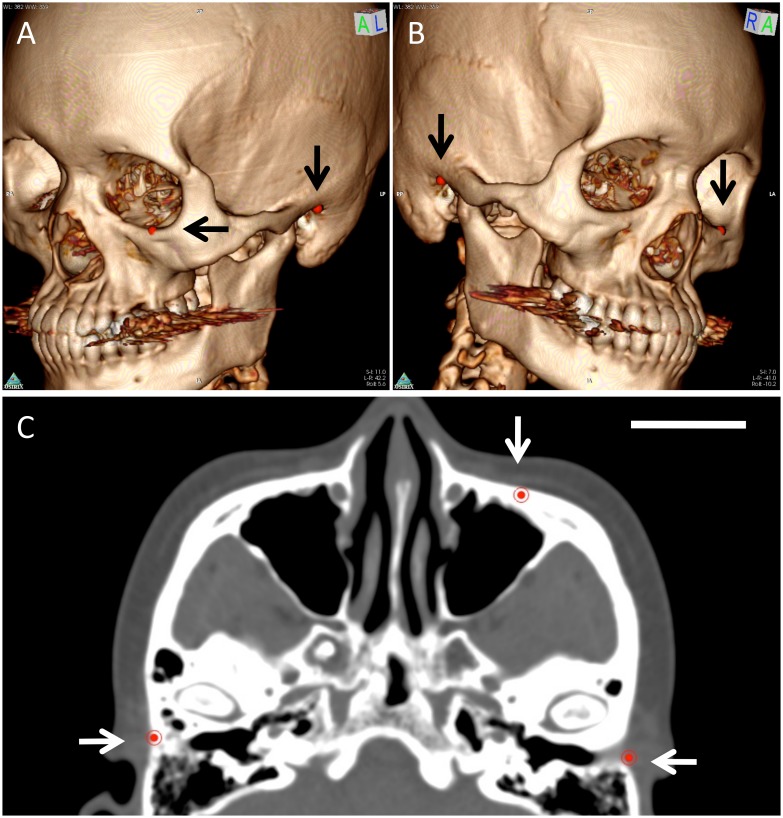
Frankfurt reorientation of the original CT DICOM data. After identifying the orbitale and porions on the 3D volume rendered skulls (black arrows, Fig. 1A and 1B), the Multiplanar Reconstruction function was used at 1mm slick thickness to identify the axial oriented slice that included the three defining points (white arrow) of the Frankfurt plane (Fig. 1C, scale bar = 3cm). The Multiplanar Reconstruction tool (not shown) can be used to identify any desired plane according to 3D landmarks using this function.

Orbital volume using ROI function was successfully calculated in the patients using OsiriX. The methodology is described below:

Frankfurt plane was located by using the 3D Multiplanar Reconstruction (3D MPR) volume rendering tool and 3D point function of OsiriX. Briefly, the built-in 3D Volume Rendering tool was used for 3D image reconstruction of the original 2D dataset. The three defining points of Frankfurt plane were identified on the 3D volume rendered image and labeled with 3D points (Fig. 1A and 1B). Then, the axial slice containing all three points (displayed automatically on corresponding 2D slices by OsiriX) was identified on the 2D image, three-plane view of 3D MPR function at 1mm slice thickness (Fig. 1C). This slice was automatically propagated caudally and cephalically using the Export function to Dicom files at 1mm slice thickness and interval, and the reoriented series was saved to a new DICOM file for further volume analysis.

**Fig 2:**
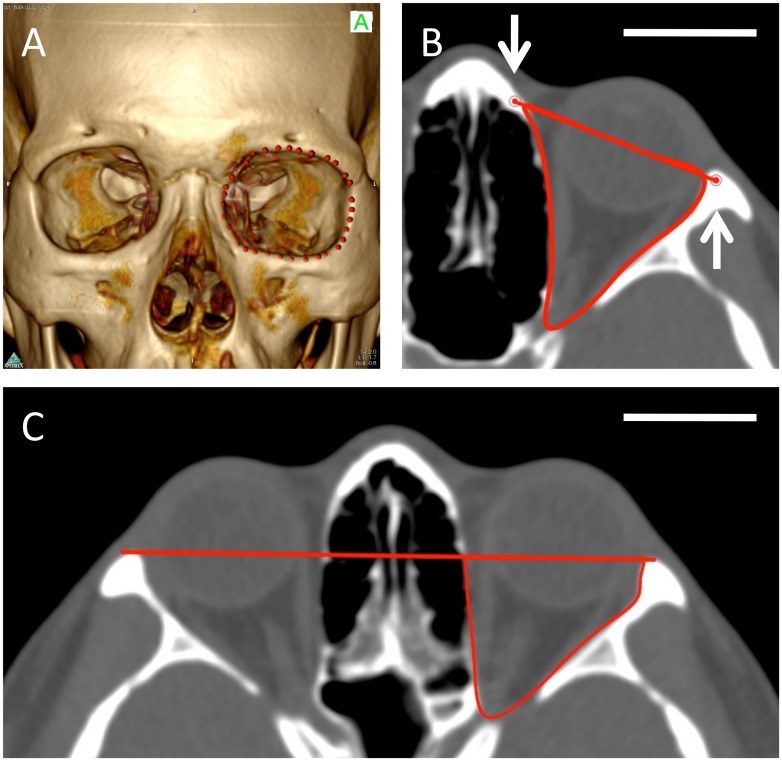
3D-assisted volume quantification of the orbit. To address the difficulty in identifying anterior limits of the orbit, the 3D Volume Rendering tool and the 3D Point tool can be used to help identify and mark the orbital rim (Fig. 2A). These 3D points can then be identified on the reoriented Frankfurt plane axial view (white arrows, Fig. 2B). Closed Polygon tool was used in this study to mark the region of interest (ROI) for sequential slices and volume reconstruction (Fig. 2B, method one). Method two utilizes the bilateral lateral orbital margins as landmarks, and defines the anterior limit at the line connecting the two sides (Fig. 2C). The Closed Polygon tool was also used to mark the ROI (Fig. 2F). (scale bar = 3cm)

**Fig 3:**
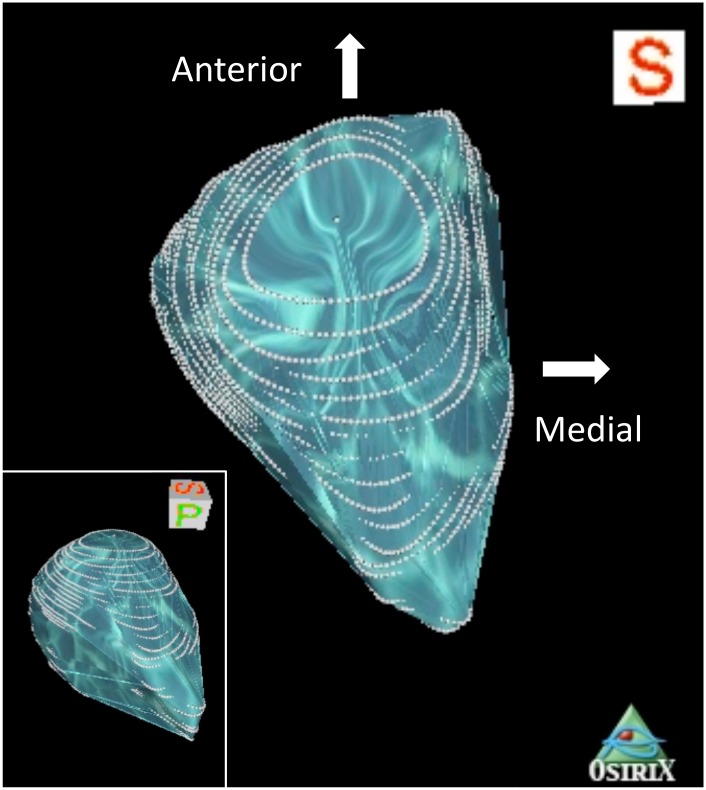
Superior view of reconstructed left orbit, method one, using OsiriX and posterior-lateral view (inset). The Calculate Volume Tool for ROI was used to reconstruct the 3D ROI and quantify the volume of the orbit. The white-dotted concentric perimeters correspond to the 2D ROIs marked by the Closed Polygon Tool. The classical pyramidal shape of the orbit can be appreciated in these two views. Orientation cubes are included to represent the viewing angles (S: superior, P: posterior).

Orbital volume estimation method one: 3D Volume Rendering tool was used to produce a 3D rendered image of the 2D data set. The bony orbital rim was outlined using the 3D point tool (Fig. 2A). This included the zygomatico-frontal processes at the lateral side, the anterior lacrimal crest at the infero-medial orbital rim, the nasal process of the frontal bone at the supero-medial side, and the supra- and infra- orbital rims. Based on these landmarks, manual segmentation with the closed polygon ROI tool was used on the 2D axial view to delineate the boundaries of the orbit. The landmarks identified as the corresponding 3D points were visible on the 2D slices (Fig. 2B). The anterior limit was defined by a line connecting the lateral and medial orbital rim landmarks on each slice [10]. The posterior limit was set at the opening of the optic foramen into the orbit. The most superior and inferior axial slices could be confirmed via sagittal plane auto-location on a sagittal-plane view. The optic canal, and soft tissue and portions of the globe protruding out of the orbital rim were excluded from volume calculation (Fig. 2B). After completing the ROIs on consecutive slices, the compute ROI volume tool was used to automatically calculate the volume of the total selected regions (Fig. 3).

Orbital volume estimation method two: The anterior limit of volume calculation was defined as a line connecting the bilateral lateral orbital margins (Fig. 2C) [11]. The volume was then calculated under the same conditions as method one, with the variation lying in the definition of the anterior border (Fig. 2C). 3D reconstruction and 3D point assistance tool was not required.

**Fig 4:**
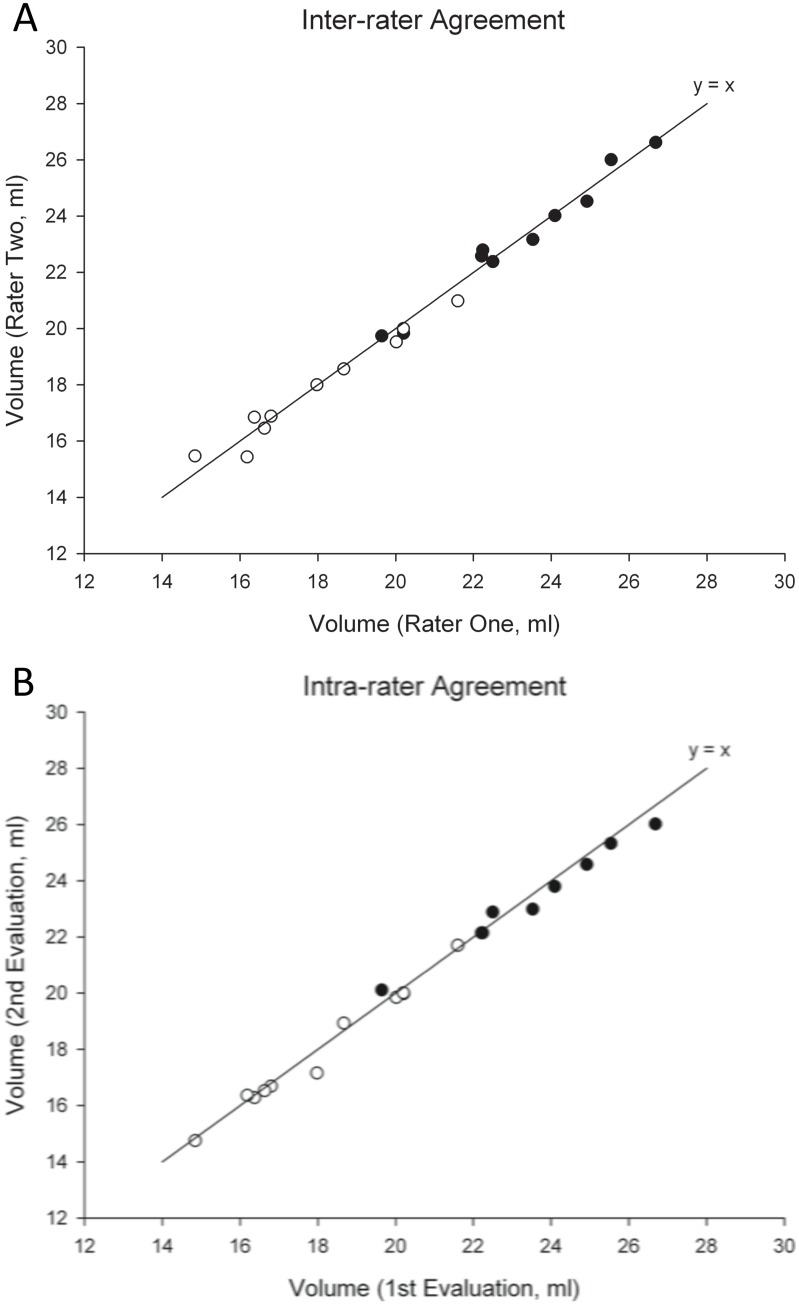
Analysis of inter-rater agreement (Fig. 4A) and intra-rater agreement (Fig. 4B) using Method One and Method Two versus line of equality (y = x). The left orbits of 10 patients were evaluated by either two raters or the same rater twice (n = 10). Black dots refer to evaluations using Method One. White dots refer to Methods Two.

**Fig 5:**
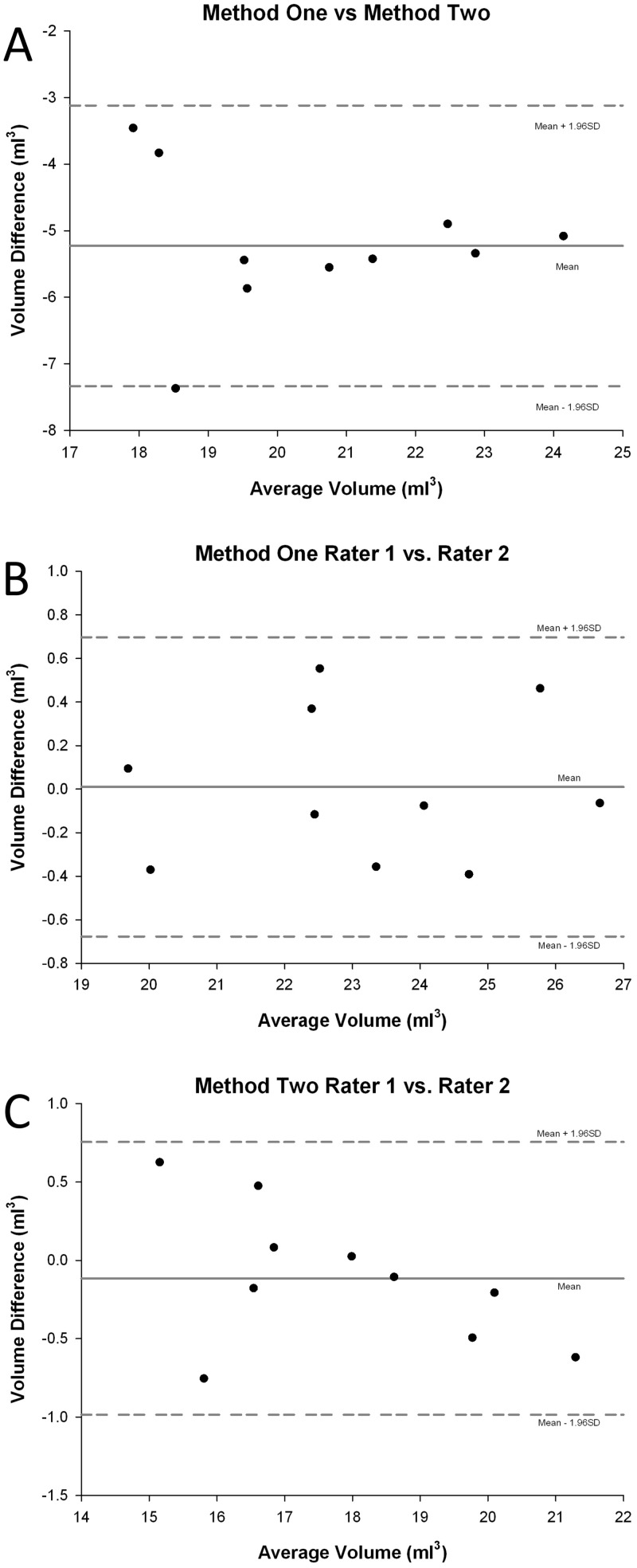
Bland-Altman analysis. Bland-Altman analysis for comparisons between methodology (Fig. 5A) and interobserver variability (Fig. 5B/5C) of outlining total left orbital volume using OsiriX. Horizontal solid line indicates mean average between methods or raters. Horizontal dashed lines indicate 95% limits of agreements (mean ± 1.96 SD).

The results of orbital volume for method one and method two are summarized in Table 1. There was a good correlation between the left and right orbital volumes. The mean absolute difference between left and right orbital volumes for each individual was 0.6±0.33 ml for method one and 0.6±0.27 ml for method two (n = 20 patients, method one range: 0.04~1.36 ml; n = 20, method two range: 0.09~1.2 ml). The mean size discrepancy, calculated as the difference between orbits divided by the lesser orbital volume within a patient, was 3.1±2.03% for females (n = 10) and 2.7±1.32% for male subjects (n = 10) according to method one. The mean size discrepancy was 3.5±1.60% for females (n = 10) and 3.3±1.58% for males according to method two. A significant inter-gender difference was noted for both left and right orbital volumes (p<0.001). Inter-rater and intra-rater volume calculations demonstrated a high level of accuracy (Fig. 4). The mean absolute difference between rater one and rater two was less than 1 ml for all cases evaluated. Mean absolute difference of repeat testing by rater one was also less than 1 ml for all cases evaluated (Table 2). The significant mean difference of results between method one and method two (n = 40 orbits) was 5.24±0.81 ml (p<0.0001). Finally, Bland-Altman analysis demonstrated a systemically lower volume calculation for method two compared to method one (Fig. 5A), while inter-rater variability was clinically insignificant (Fig. 5B and 5C).

**Table 1:** Statistical Analysis of Orbital Volume (ml) in Taiwanese Patients (n = 20).

	Left	Right	Absolute Difference	% Diff.	R value
Method One					
Male (n = 10)	24.3±1.51	24.7±1.17	0.6±0.30	2.7±1.32	0.92
Female (n = 10)	21.0±1.21	21.1±1.30	0.6±0.38	3.1±2.03	0.82
All (n = 20)	22.7±2.14	22.9±2.19	0.6±0.33	2.9±1.68	0.95
Method Two					
Male (n = 10)	19.0±1.68	19.1±1.45	0.6±0.29	3.3±1.58	0.91
Female (n = 10)	16.0±1.01	16.1±0.92	0.6±0.25	3.5±1.60	0.79
All (n = 20)	17.5±2.05	17.6±1.95	0.6±0.27	3.4±1.55	0.95

The mean left and right orbital volumes for subjects and percentage difference between left and right sides are presented. Pearson’s correlation was used to evaluate the relationship between the two sides, and the R values are reported (p<0.05 for all evaluations).

**Table 2:** Inter-rater and Intra-rater measurement accuracy (n = 10).

	Absolute difference (ml)	Difference range (ml)
Inter-rater M1	0.29±0.180	0.06~0.55
Inter-rater M2	0.36±0.266	0.03~0.75
Intra-rater M1	0.33±0.191	0.07~0.54
Intra-rater M2	0.21±0.220	0.09~0.82

The mean absolute difference and difference range is considered acceptable for orbital volume calculations between raters and during repeat testing on the left orbit (n = 10). (M1 = method one, M2 = method two)

## Discussion

The difficulty in evaluating the orbital volume lies in its complicated anatomy. The orbit is pyramidal shaped, with numerous foramina and openings. The anterior limit of the orbit is characterized by numerous bony ridges, such as the supraorbital notch and the anterior lacrimal crest. This is a source of variation in the evaluation of orbital volume in patients, as the anterior opening of the orbit does not lie within a single plane [7, 13]. Image orientation is a key factor for accurate volume analysis utilizing additive methodologies with serial ROIs. Usually, 2D slice orientation is skewed due to improper head positioning during CT scanning [14]. In the acute trauma setting, it is difficult to standardize the patient’s position. Hence, it is necessary to re-orient 2D CT data sets, a function provided by the current imaging platform. To minimize measurement errors, slice orientation was standardized using the 3D MPR function to the reference Frankfurt horizontal plane. Using OsiriX’s built-in functions, this was a convenient process that usually required less than 2 minutes of time.

The mean difference of the Bland-Altman plot indicates systematic error [15]. Method two, which does not account for a significant portion of the anterior-medial aspect of the orbit, produced systemically lower amount of orbital volume. However, it is less time consuming and doesn’t require the use of 3D points to mark the orbital margin since the margin of lateral orbital rims are easy to identify. The methodology of volume measurement should take into consideration the case at hand. For example, Ji et al.’s method, which included the entire globe and protruding soft tissue, could be useful for evaluating cases in which external soft tissue volume is also important [7]. For both of the methods used, the inter-rater variability was clinically insignificant according to Bland-Altman analysis (Fig. 5A), and the distribution of the volume differences demonstrated no biases between the raters when using the OsiriX tool for volume assessment (Fig. 5B and 5C). This is important because manual segmentation is often limited by differences in bony landmark determination between raters, leading to biased results where one rater constantly has larger calculation outcomes [16]. Bland-Altman analysis provides further evidence of the benefits and reliability of using 3D point-assisted landmark determination and reorienting of CT slices.

The reported orbital volume in adult Taiwanese patients from this study for left and right orbits was found to be 24.3±1.51 ml and 24.7±1.17 ml in male patients and 21.0±1.21 ml and 21.1±1.30 ml in female patients according to method one (Table 1). The anatomical parameters of method one are more feasible for clinical use as they match the anatomical limits of the gold standard for orbital volume measurement. The gold standard is the fluid or sand displacement method [17, 18]. The first report of orbital volume in Northern Chinese male subjects via sand filling dates back to 1933, in which orbital volume was found to be 29.3±2.5 ml [19]. The reason for this considerably larger result for Chinese subjects is unknown, but could be related to ethnicity or preservation methods of the skulls used. Although modern technology allows detailed CT scans of patient to be rapidly 3D printed into skull models, which can then be subjected to the gold standard method, it is still impractical to use this method for orbital volume estimation in patients [20]. Hence, the CT based reconstruction method is considered to be the most feasible methodology for orbital volume estimation.

This current study is considered to be the first report of CT-based normal orbital volume in Taiwanese Chinese patients using the anatomical parameters corresponding to the gold standard with a detailed CT protocol. There have been a few studies on the Chinese orbit. One CT study on Chinese patient conducted by Ji, et al. defined an anterior limit including the entire globe and eyelid tissue [7]. Possibly due to the extra-orbital contents, their results approximated those of Caucasian populations (28.41±2.09 ml) and were higher than a number of studies in Asian populations (Table 3) [6, 8, 9, 21]. The study on Hong Kong Chinese patients by Chau et al. utilized MRI images for orbital volume calculation. However, differences exist between CT and MRI imaging techniques and CT is more frequently used for the evaluation of craniofacial deformities or facial bone fractures than MRI. Finally, a Chinese report by Chen et al. using a 5mm CT slice thickness and distance protocol reported the orbital volume of five different age groups [22]. In Chen’s study, the use of reconstructed 3 mm slices for orbital estimations based on Cavalieri’s principle is not precise enough due to the irregularity of the orbital anatomy. This may have led to the larger orbital volume results reported (Table 3). As a result, a more detailed CT protocol has been used in the current study.

**Table 3:** Orbital volume of Asians reported in the literature.

Author	Orbital Volume (ml)	Ethnicity	Imaging Modality	Notes
Ji et al.	Male: 26.04±2.6	Chinese	CT	Included extraorbital contents
	Female: 23.32±1.87			
Furuta M.	Male: 23.06±2	Japanese	CT	
	Female: 20.9±1.3			
Chau et al.	Male: 22.2±1.38	Hong Kong Chinese	MRI	OSIRIS software
	Female: 19.81±2.23			
Kim et al.	Male: 21.5±1.72	Korean	MRI	BrainVoyager software
	Female: 19.47±1.84			
Chen et al.	Male: 25.04±2.37	Chinese	CT	5mm CT slice thickness/distance protocol
	Female: 22.89±2.67			
Shyu et al.	Method One:	Taiwan Chinese	CT	OsiriX MD software
	Male: 24.5±1.33			
	Female: 21.1±1.22			
	Method Two:			
	Male: 19.1±1.53			
	Female: 16.0±0.94			

Reported values of orbital volume for Asian ethnic groups in the literature are summarized.

As reported in literature, there is no significant difference between the left and right orbits within individuals [3, 6, 23]. Our study confirms this finding in Taiwan Chinese patients, with a high correlation and small size difference between left and right orbital volume. However, an intra-individual size discrepancy up to 8% has been reported in the literature [24]. In our study, the largest difference calculated was 7.5% using method one, but was limited to one case; all other cases had less than 5% discrepancy. With careful selection, the use of one orbit as a control when operating on the other seems feasible.

In this study, the use of open-source OsiriX imaging software by non-radiologists was evaluated. Its usefulness in the preoperative surgical setting was demonstrated. The combination of orbital volume information and other parameters such as bony angles and symmetry will allow OsiriX to be used as a navigational tool during surgery [25]. In other studies, relationships between orbital volume change, soft tissue volume, and enophthalmos or exophthalmos have been reported, expanding the function of orbital volume calculations as a prognostic tool in the orbital fracture scenario [26, 27]. One weakness to the use of OsiriX is that the software can only be executed on a Macintosh system. Additionally, there is a learning curve to speed up ROI drawing for orbital volume calculation. The differences between Chinese populations globally may exist and the absolute volume measurements may not correlate well across populations. However, drawing from conclusions across the literature, it could be safe to utilize the normal orbit as a control for management of the diseased side.

## Conclusion

The use of 3D assisted landmark for reorientation of 2D slices and ROI tool in OsiriX produced highly reproducible inter-rater and intra-rater results with either method one or two. OsiriX is an accessible and simple personal tool for pre-operative assessment in regards to orbital surgery.

## Supporting Information

S1 Dataset Dataset of orbital volume calculations conducted in this study. (XLSX)
